# Effects of Poly(ethylene-*co*-glycidyl methacrylate) on the Microstructure, Thermal, Rheological, and Mechanical Properties of Thermotropic Liquid Crystalline Polyester Blends

**DOI:** 10.3390/polym12092124

**Published:** 2020-09-17

**Authors:** Sang Hoon Lee, Ha-Bin Jeon, Gyu-Hyun Hwang, Young Seung Kwon, Ji-Su Lee, Gyu-Tae Park, Soo-Yeon Kim, Ha-Eun Kang, Eun-Ji Choi, Sun-Hwa Jang, Youn Eung Lee, Young Gyu Jeong

**Affiliations:** 1 Department of Applied Organic Materials Engineering, Chungnam National University, Daejeon 34134, Korea; skyhoon1202@naver.com (S.H.L.); jeon3704@naver.com (H.-B.J.); hgh9040@naver.com (G.-H.H.); dudtmd06@naver.com (Y.S.K.); jeesoo1338@naver.com (J.-S.L.); dlrbqkr@naver.com (G.-T.P.); suyeon8806@naver.com (S.-Y.K.); khe9895@naver.com (H.-E.K.); eji6936@naver.com (E.-J.C.); 2 Seyang Polymer Co., Ltd., Anseong-si, Gyeonggi-do 17602, Korea; shjang@seyangpolymer.com (S.-H.J.); ylee@seyangpolymer.com (Y.E.L.)

**Keywords:** thermotropic liquid crystalline polyester, polyethylene copolymer, rheological property, thermal stability, mechanical property

## Abstract

In this study, a series of thermotropic liquid crystalline polyester (TLCP)-based blends containing 1–30 wt% poly(ethylene-*co*-glycidyl methacrylate) (PEGMA) were fabricated by masterbatch-assisted melt-compounding. The scanning electron microscopy (SEM) images showed a uniformly dispersed microfibrillar structure for the TLCP component in cryogenically-fractured blends, without any phase-separated domains. The FT-IR spectra showed that the carbonyl stretching bands of TLCP/PEGMA blends shifted to higher wavenumbers, suggesting the presence of specific interactions and/or grafting reactions between carboxyl/hydroxyl groups of TLCP and glycidyl methacrylate groups of PEGMA. Accordingly, the melting and crystallization temperatures of the PEGMA component in the blends were greatly lowered compared to the TLCP component. The thermal decomposition peak temperatures of the PEGMA and TLCP components in the blends were characterized as higher than those of neat PEGMA and neat TLCP, respectively. From the rheological data collected at 300 °C, the shear moduli and complex viscosities for the blend with 30 wt% PEGMA were found to be much higher than those of neat PEGMA, which supports the existence of PEGMA-*g*-TLCP formed during the melt-compounding. The dynamic mechanical thermal analysis (DMA) analyses demonstrated that the storage moduli of the blends decreased slightly with the PEGMA content up to 3 wt%, increased at the PEGMA content of 5 wt%, and decreased again at PEGMA contents above 7 wt%. The maximum storage moduli for the blend with 5 wt% PEGMA are interpreted to be due to the reinforcing effect of PEGMA-*g*-TLCP copolymers.

## 1. Introduction

Thermotropic liquid crystalline polyesters (TLCPs)—aromatic polyesters exhibiting liquid crystallinity in a molten state—are used in a variety of industrial sectors because of their excellent thermal stability, dimensional stability, mechanical performance, and chemical resistance [1,2,3,4,5,6,7,8]. Since TLCPs can be processed easily due to their low viscosity as a result of their ordered melt, an extremely high molecular orientation can be attained in fibers, films, and injection molded parts. The combination of excellent physical properties and facile melt-processability makes TLCPs an ideal engineering material for high-end applications in automobile, optical, and electronic devices. Despite their outstanding thermal and mechanical performance, they are expensive in terms of the cost for expanding practical applications. To achieve a higher price-performance ratio without compromising the excellent thermal and mechanical properties of TLCPs, blending with conventional thermoplastic polymers is considered the most economical and effective method.

The blending of TLCPs with commercial thermoplastics, such as polyethylene (PE), polypropylene (PP), polycarbonate (PC), poly(ethylene terephthalate) (PET), etc., has been known to lead to viscosity reductions, an improved processability, and enhanced mechanical and barrier properties [9,10,11,12,13,14,15,16]. Chan et al. investigated the flow-induced chain alignment and disentanglement of TLCP/high density polyethylene (HDPE) blends and proposed a mechanism elucidating the viscosity reducing effects of the incorporation of TLCP as a processing aid into the HDPE matrix [17]. They found that TLCP droplets firstly deform into long fibrils during entry flow, which is followed by the alignment of stiff TLCP chains within the TLCP droplets, and such an alignment of TLCP chains forces neighboring HDPE chains to align and disentangle, eventually inducing a reduced bulk viscosity of blends. Qin et al. investigated the morphological structures and tensile mechanical properties of TLCP/PP blend fibers [10]. It was found that the tensile mechanical properties of the as-spun fibers could be improved upon the addition of TLCPs. Che et al. reported the influences of phosphorous-containing TLCP on the transesterification-controlled compatibility, microfibrillation, and rheological and mechanical properties of PC blends with acrylonitrile butadiene styrene (ABS) terpolymer [14]. They found that an optimal extent of transesterification between TLCP and PC is critical to enhancing the compatibility and tensile mechanical properties. The structure–process–property relationship of TLCP/PET blends has also been investigated. It was reported that TLCP accelerates the overall crystallization rate of PET [18]. The addition of TLCP was also found to contribute to the enhancement of the molecular orientation, crystallite size, and mechanical properties of PET blend fibers [19]. However, in the above reports on TLCP/thermoplastics blends, TLCPs served as a processing aid or reinforcing agent for improving the thermal and mechanical properties of thermoplastic matrices as major components.

In this study, we tried to investigate the structure–property relationship of TLCP-dominant blends containing thermoplastic as a minor component. PE is a thermoplastic polymer widely used in various fields because of its excellent flexibility, processability, and cost-efficiency. The blending of TLCP with PE can achieve enhanced mechanical properties in terms of the toughness and an acceptable performance-cost ratio. However, TLCP/PE blends are known to be immiscible, resulting in poor interfacial adhesion [17,20]. Immiscibility and incompatibility can induce low mechanical properties, such as a low tensile strength, impact strength, and modulus, in polymer blends. Incorporating reactive functional groups into the polymer is an easy way of increasing the compatibility of the polymer-polymer interface [21]. Polymers with reactive functional groups can form in-situ graft or block copolymers with another polymer during the blending process [22,23]. Accordingly, poly(ethylene-*g*-glycidyl methacrylate) (PEGMA) has been considered as a reactive polymer component in polymer blend systems [24,25]. For the purpose of this study, we fabricated a series of TLCP-dominant blends, including 1–30 wt% PEGMA loadings, by a facile masterbatch-based melt-compounding method for the first time. The morphological, microstructural, and crystalline features of melt-compounded TLCP/PEGMA blends were investigated by using scanning electron microscopy (SEM), Fourier-transform infrared (FT-IR) spectroscopy, and the X-ray diffraction method (XRD), respectively. The thermal transition behavior and thermal stability of the blends were characterized by using differential scanning calorimetry (DSC) and thermogravimetric analysis (TGA), respectively. The rheological properties of the blends in a melt state were measured under a small amplitude dynamic oscillatory shear. The temperature-dependent mechanical properties of TLCP/PEGMA blends were investigated by using dynamic mechanical thermal analysis (DMA). The morphological features; microstructures; and rheological, thermal, and mechanical properties of TLCP/PEGMA blends were discussed in terms of the PEGMA content, intermolecular interaction, and reactivity between the PEGMA and TLCP in the blends.

## 2. Experimental

### 2.1. Materials

TLCP, which is composed of 27 mol% hydroxybenzoic acid (HBA) and 73 mol% hydroxynaphthoic acid (HNA), was kindly supplied by Seyang Polymer Com., Incheon, Korea. PEGMA (IGETABOND, Sumitomo Chemical Com., Tokyo, Japan) containing 6 wt% GMA, with a mass flow rate of 3 g/10 min at 190 °C, density of 0.931 g/cm^3^, melting point of 105 °C, glass transition temperature of −26 °C, tensile strength at break of 18 MPa, elongation at break of 650%, and modulus = 1000 kg/cm^2^, was used as a thermoplastic polymeric modifier of TLCP.

### 2.2. Preparation of TLCP/PEGMA Blends

**Figure 1:**
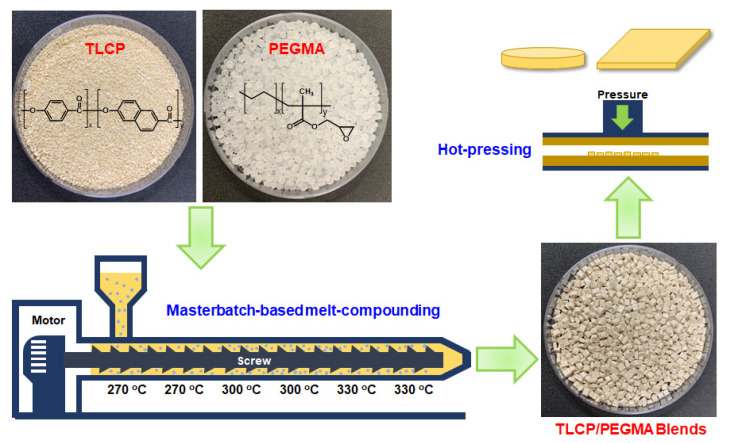
A schematic procedure for fabricating thermotropic liquid crystalline polyester (TLCP)/poly(ethylene-*g*-glycidyl methacrylate) (PEGMA) blends via masterbatch-based melt-compounding.

TLCP-based blends with 1–30 wt% PEGMA loadings were fabricated by masterbatch-based melt-compounding, as represented schematically in Figure 1. Before melt-compounding, TLCP and PEGMA chips were dried at 60 °C in vacuo for 24 h to minimize the effects of residual moisture. To improve the compatibility of PEGMA and TLCP, a masterbatch composed of 70 wt% TLCP and 30 wt% PEGMA was prepared by melt-compounding with the aid of a twin-screw extruder (BA-11, Bautek Com.) at a screw speed of 50 rpm. The temperature profile of the heating zone from the hopper to die was set to be 270, 270, 300, 300, 330, and 330 °C. The masterbatch extrudates were cooled in a water bath and pelletized into chips, which were then dried at 60 °C in vacuo for 12 h. For fabricating a series of TLCP/PEGMA blends with different PEGMA loadings of 1–30 wt%, predetermined amounts of masterbatch chips were melt-compounded with neat TLCP chips in the same condition, cooled in a water bath, pelletized into chips, and dried at 60 °C in vacuo for 12 h. The PEGMA content in the final TLCP/PEGMA blends was controlled to be 1, 2, 5, 7, 10, 20, and 30 wt%, respectively. The final TLCP/PEGMA blends were named TLCP-Ex, where x denotes the content of PEGMA by weight percent (wt%). For the structure and property characterization, TLCP/PEGMA sheets with a 0.5 mm thickness were fabricated by melt-pressing in a hot press at 310 °C for 5 min, followed by natural cooling to room temperature.

### 2.3. Characterization

The morphological features of TLCP/PEGMA blends were characterized by using a scanning electron microscope (SEM, ZEISS, Merlin compact, Oberkochen, Germany) operating at the accelerating voltage of 8 kV. For obtaining cross-sectional SEM images, neat TLCP and its blends were fractured in a liquid nitrogen bath and then coated with platinum in a vacuum sputtering chamber for 60 s.

The crystalline features of neat TLCP, neat PEGMA, and TLCP/PEGMA blends were identified by using an X-ray diffractometer (D8 DISCOVER, Bruker AXS, Karlsruhe, Germany) with Cu-Kα radiation (λ = 0.15418 nm) in the 2θ range of 5–50°. 

The chemical structures and molecular interactions of TLCP/PEGMA blends and their components were analyzed by using a FT-IR spectrometer (Nicolet iS10, Thermo Fisher Scientific, Waltham, MA, USA).

The thermal transition properties of neat TLCP, neat PEGMA, and TLCP/PEGMA blends were investigated with a differential scanning calorimeter (DSC6000, PerkinElmer Inc., Waltham, MA, USA) over the temperature range of 30–300 °C at a heating and cooling rate of 10 °C/min under nitrogen atmosphere.

The thermal stability and thermal degradation behavior of TLCP-based blends with different PEGMA contents were analyzed by using a thermogravimetric analyzer (TGA4000, PerkinElmer Inc., Waltham, MA, USA) in the temperature range of 30–800 °C at a heating rate of 10 °C/min under nitrogen atmosphere.

The rheological properties of neat TLCP, neat PEGMA, and TLCP/PEGMA blends under an oscillatory dynamic shear were measured by using a rheometer (MCR102, Anton Paar, Graz, Austria) with a parallel plate geometry with a 25 mm diameter and 1 mm gap. Dynamic frequency sweep measurements were performed at 300 °C in the frequency range of 0.1–100 rad/s.

The temperature-dependent dynamic mechanical properties of TLCP/PEGMA blends were characterized by using a dynamic mechanical analyzer (DMA8000, PerkinElmer Inc., Waltham, MA, USA) with a single cantilever geometry at a frequency of 0.1 Hz. The DMA experiments were carried out in the temperature range from −50 to 240 °C at a heating rate of 5 °C/min.

## 3. Results and Discussion

### 3.1. Morphological and Structural Characterization

**Figure 2:**
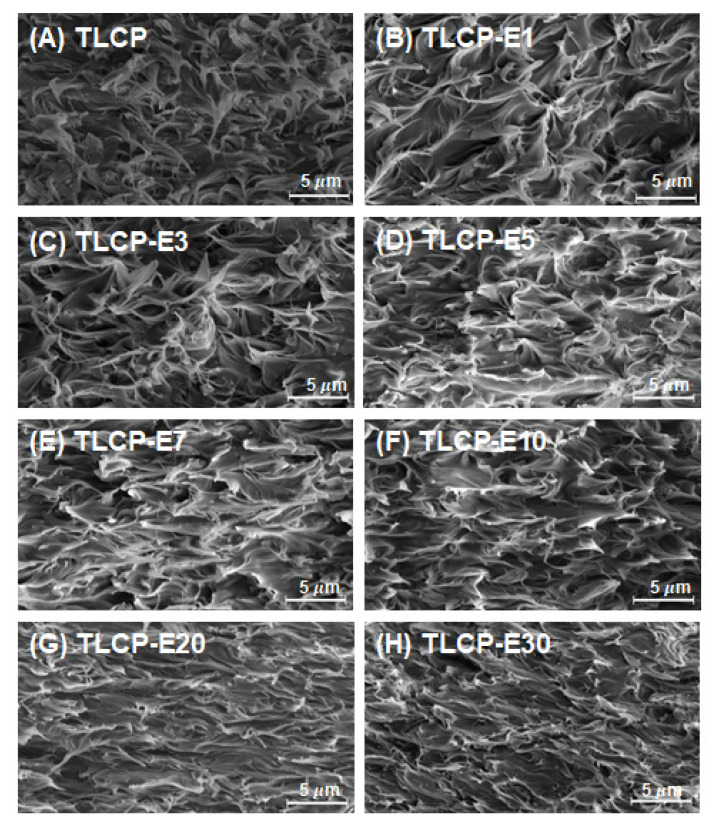
Cross-sectional scanning electron microscopy (SEM) images of neat TLCP and TLCP/PEGMA blends: (**A**) TLCP; (**B**) TLCP-E1; (**C**) TLCP-E3; (**D**) TLCP-E5; (**E**) TLCP-E7; (**F**) TLCP-E10; (**G**) TLCP-E20; (**H**) TLCP-E30.

In order to characterize the morphological features of TLC/PEGMA blends, SEM images of the fractured surfaces of TLCP and TLCP/PEGMA blends were obtained, as shown in Figure 2. In the case of neat TLCP, microfibrils with a highly orientated structure can be observed over the fractured cross-section due to the rigid backbone chains of TLCP. For TLCP/PEGMA blends, the cross-sectional morphological features are almost identical to those of neat TLCP, without showing apparent phase-separated domains, which suggests good compatibility between TLCP and PEGMA.

**Figure 3:**
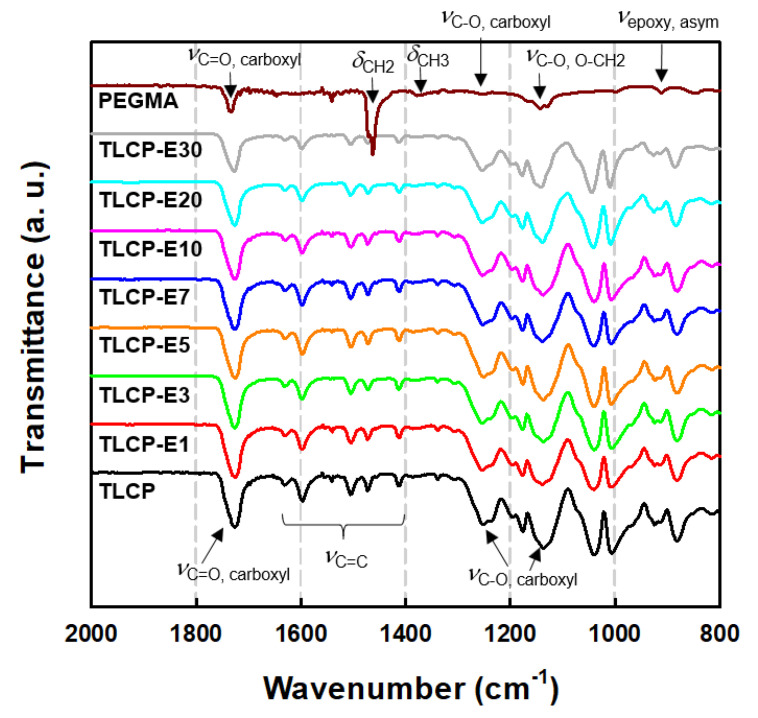
FT-IR spectra of neat TLCP, neat PEGMA, and their blends with different PEGMA contents of 1–30 wt%.

**Figure 4:**
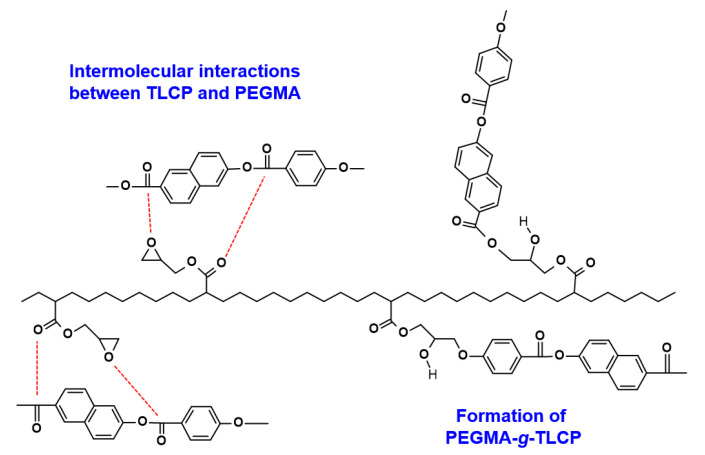
Schematic drawing for the formation of PEGMA-*g*-TLCP copolymers and the intermolecular interactions between TLCP and PEGMA in the blends.

To identify the chemical structures and molecular interactions of TLCP and PEGMA, the FT-IR spectra of the neat TLCP, neat PEGMA, and TLCP/PEGMA blends were obtained, as can be seen in Figure 3. For neat TLCP, the characteristic bands associated with C=C stretching vibrations of the naphthalene and benzene rings in the backbone can be detected in the range of 1400–1650 cm^−1^. In addition, characteristic C=O stretching (*ν*_C=O, carboxyl_) and C–O stretching (*ν*_C–O, carboxyl_) vibrations of the carboxyl group can be observed at ~1724 and ~1249/1178 cm^−1^, respectively [26,27,28]. In the case of neat PEGMA, an asymmetric stretching vibrational band of the epoxy ring (*ν*_epoxy, asym_) can be observed at 908 cm^−1^. In addition, characteristic vibration bands related to the C–O stretching of O–CH_2_ (*ν*_C–O, O–CH2_), C–O stretching of the carboxyl group of GMA (*ν*_C−O, carboxyl_), symmetric deformation of the methyl group (δ_CH3_), bending deformation of the methylene group (δ_CH2_), and C=O stretching of carboxyl groups of GMA (*ν*_C=O, carboxyl_) were detected at 1141, 1234, 1370, 1463, and 1733 cm^−1^, respectively [29,30]. For the blends with 1–30 wt% PEGMA loadings, all of the FT-IR spectra were quite similar to those of neat TLCP. For a quantitative comparison, the characteristic vibrational bands associated with carboxyl groups are summarized in Table 1. It can be seen that the C–O stretching bands (*ν*_C–O, carboxyl_) at 1176 and 1249 cm^−1^ for neat TLCP were shifted to 1179 and 1253 cm^−1^ for the TLCP-E30 blend, as the PEGMA content increased in the blends. In addition, the C=O stretching band (*ν*_C=O, carboxyl_) at 1724 cm^−1^ for neat TLCP was shifted to 1727 cm^−1^ for the TLCP-E30 blend. These results demonstrate that intermolecular interactions and/or a chemical reaction might exist between carboxyl/hydroxy groups of TLCP and epoxy/methacrylate groups of PEGMA [31], as represented schematically in Figure 4.

**Figure 5:**
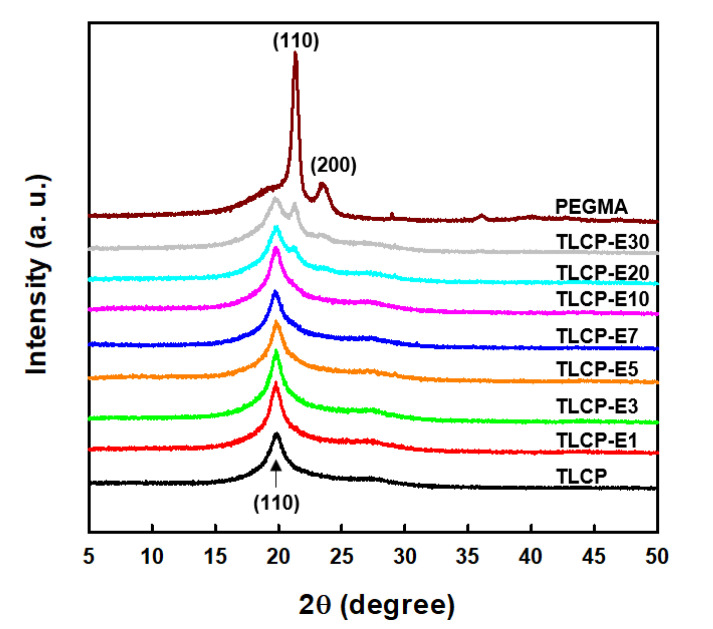
X-ray diffraction patterns of neat TLCP, neat PEGMA, and TLCP/PEGMA blends.

To characterize the crystalline features of TLCP/PEGMA blends, X-ray diffraction patterns of neat TLCP, neat PEGMA, and their blends were obtained, as can be seen in Figure 5. For neat TLCP, a typical diffraction peak at ~19.8°, which corresponds to *d*-spacing of 4.5 nm based on Bragg’s law, was observed and this is associated with the (110) plane of the TLCP crystalline structure [4,32]. In the case of PEGMA, strong diffraction peaks can be observed at 21.3 and 23.3°, which correspond to the (110) and (200) planes of PE crystals, respectively [33]. For all TLCP/PEGMA blends, the strong diffraction peak for TLCP crystals can be observed at 19.8°, whereas the characteristic diffraction peaks at 21.3 and 23.3° of PE crystals can only be detected for the blends with more than 10 wt% PEGMA. This result demonstrates that the PEGMA and TLCP in the blends do not noticeably affect each other’s crystalline structure, although their relative diffraction intensities decrease when increasing the counterpart content in the blends. In particular, the crystalline diffraction peaks of the PEGMA component decrease significantly in the blends, which might be caused by the restricted crystallization of PEGMA in the blends owing to the formation of graft copolymers (PEGMA-*g*-TLCP) and the intermolecular interactions between TLCP and PEGMA components (Figure 4).

### 3.2. Thermal Transition and the Decomposition Property

**Figure 6:**
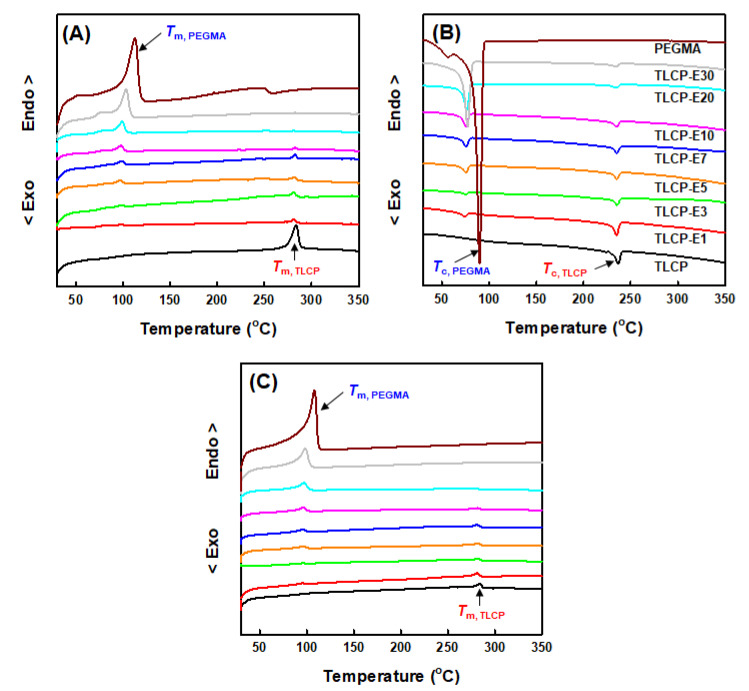
DSC thermograms of neat TLCP, neat PEGMA, and TLCP/PEGMA blends: (**A**) First heating; (**B**) first cooling; (**C**) second heating.

**Figure 7:**
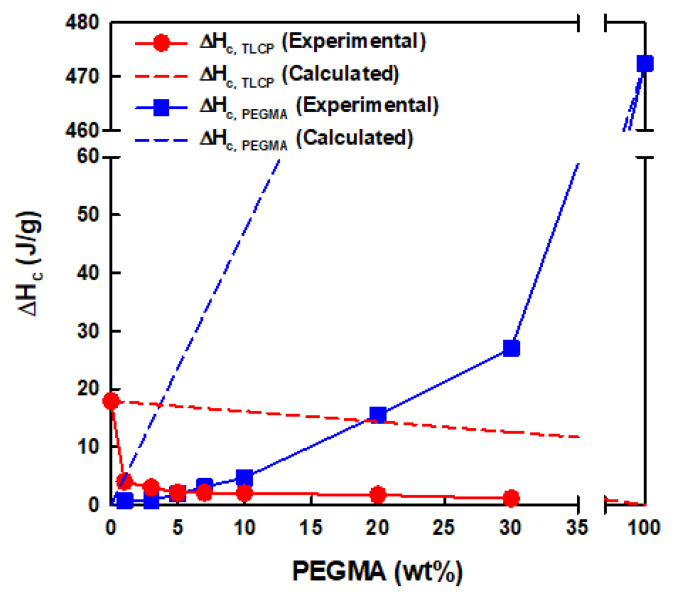
Experimental and calculated crystallization enthalpies (Δ*H*_c, TLCP_ and Δ*H*_c, PEGMA_) of TLCP and PEGMA components in the blends as a function of the PEGMA content.

The thermal transition behaviors of neat TLCP, neat PEGMA, and TLCP/PEGMA blends were investigated by obtaining DSC heating and cooling thermograms, as shown in Figure 6. As a result, the peak temperatures (*T*_m_ and *T*_c_) and enthalpies (Δ*H*_m_ and Δ*H*_c_) of the melting and crystallization transitions of TLCP and PEGMA components in the blends were summarized and are presented in Table 2. In the first heating thermograms of Figure 6A, the *T*_m, TLCP_ and *T*_m, PEGMA_ values of neat TLCP and PEGMA are characterized as ~281 and ~110 °C, respectively. In the cases of TLCP/PEGMA blends, the *T*_m, TLCP_ values of 276–279 °C for the TLCP component are slightly lower than that of neat TLCP, whereas the *T*_m, PEGMA_ values of the PEGMA component decrease noticeably from ~101 °C of TLCP-E30 to ~85 °C of TLCP-E1 when decreasing the PEGMA content in the blends. In the first cooling thermograms of Figure 6B, the crystallization temperatures (*T*_c, TLCP_ and *T*_c, PEGMA_) of neat TLCP and PEGMA are characterized as ~237 and ~90 °C, respectively. For TLCP/PEGMA blends, the *T*_c, TLCP_ values of 235–237 °C for the TLCP component are quite comparable to that of neat TLCP, irrespective of the PEGMA content, while the *T*_c, PE_ values of 76–79 °C for the PEGMA component are far lower than that of neat PEGMA. In the second heating thermograms of Figure 6C, the melting transition behavior of TLCP/PEGMA blends is quite similar to that in the first heating thermograms. In comparison to the TLCP component with a rigid backbone chain, the significantly lowered *T*_m, PEGMA_ and *T*_c, PEGMA_ values of the PEGMA component in the blends are conjectured to be caused by the restricted crystallization rate, as well as the thin crystal formation of PEGMA with a flexible backbone chain owing to the chemical reactions and specific interactions between PEGMA and TLCP, as supported by the above SEM, FT-IR, and X-ray diffraction analyses. Consistently, the experimental melting and crystallization enthalpies (Δ*H*_m_ and Δ*H*_c_) of TLCP and PEGMA components in the blends (Table 2) were found to be much lower than the values calculated by the rule of mixtures. For instance, the crystallization enthalpies (Δ*H*_c, TLCP_ and Δ*H*_c, PEGMA_) of TLCP and PEGMA components in the blends were plotted as a function of the PEGMA content and compared with the calculated values, as can be seen in Figure 7.

**Figure 8:**
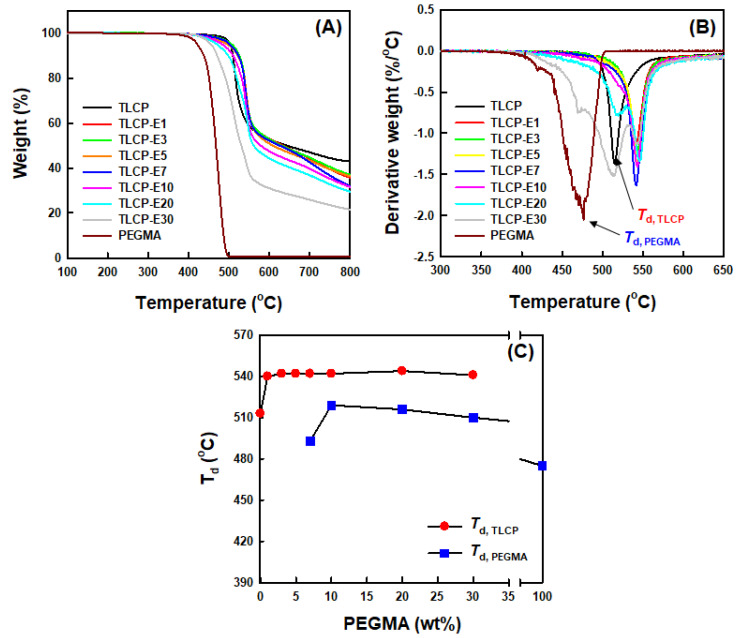
(**A**) Thermogravimetric analysis (TGA) thermograms, (**B**) DTG thermograms, and (**C**) thermal decomposition peak temperatures of neat TLCP, neat PEGMA, and TLCP/PEGMA blends.

The influence of the PEGMA content on the thermal decomposition behavior of TLCP-based blends was characterized by using TGA and DTG thermograms of neat TLCP, neat PEGMA, and TLCP/PEGMA blends, as shown in Figure 8A,B. It could be found that neat TLCP and PEGMA exhibit single-step thermal decomposition behavior, whereas the blends with 7–30 wt% PEGMA contents show two-step thermal decomposition behavior. The thermal decomposition peak temperatures (*T*_d, TLCP_ and *T*_d, PEGMA_) of TLCP and PEGMA components in the blends, which were obtained from the DTG thermograms, were plotted as a function of the PEGMA content, as can be seen in Figure 8C. The *T*_d, TLCP_ value of neat TLCP was measured to be ~513 °C and the *T*_d, PEGMA_ value of neat PEGMA was ~475 °C. For all TLCP/PEGMA blends, the *T*_d, TLCP_ values of the TLCP component were found to be in the range of 540–544 °C, which are values 17–21 °C higher than the value of neat TLCP, indicating the enhanced thermal stability of the TLCP component in the blends. The enhanced thermal stability of the TLCP component in the blends is conjectured to be due to the thermal insulation layer formation of a PEGMA component with a lower thermal stability. In addition, the *T*_d, PEGMA_ values of the PEGMA component in the blends with 7–30 wt% PEGMA contents are in the range of 493–510 °C, which are values 18–35 °C higher than that of neat PEGMA. The highly increased *T*_d, PEGMA_ values of the PEGMA component in the blends are believed to have been induced by the enhanced thermal stability of the PEGMA component that reacted or interacted with the TLCP component. It is thus reasonable to contend that the thermal stability of both TLCP and PEGMA components in the blends could be enhanced by the synergistic effects of the chemical reaction and enhanced compatibility between PEGMA and TLCP.

### 3.3. Rheological Property

**Figure 9:**
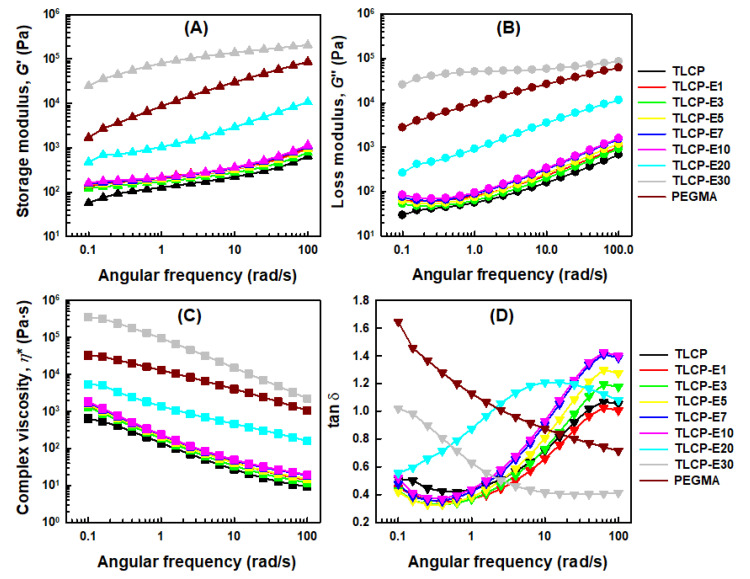
Changes of the (**A**) shear storage modulus (*G*’), (**B**) shear loss modulus (*G*”), (**C**) complex viscosity (h*), and (**D**) loss tangent (tan δ) of neat TLCP, PEGMA, and TLCP/PEGMA blends as a function of the angular frequency.

The shear storage modulus (*G*’), shear loss modulus (*G*”), loss tangent (tan ™), and complex viscosity (η*) of neat TLCP, neat PEGMA, and TLCP/PEGMA blends at 300 °C were measured as a function of the angular frequency, as can be seen in Figure 9. The shear storage moduli, shear loss moduli, and complex viscosities of neat TLCP at 300 °C were much lower than those of neat PEGMA (Figure 9A–C), which is due to the facile alignment of TLCP with a stiff chain backbone with the applied shear [5], compared to PEGMA with a high chain flexibility and entanglement in a melt state. The overall shear moduli and complex viscosities of the blends with 1–10 wt% PEGMA loadings are quite consistent with those of neat TLCP, although they increase slightly when increasing the PEGMA content in the blends. This demonstrates that the frequency-dependent shear moduli and complex viscosities of the blends are dominated by the TLCP component. On the other hand, the overall shear moduli and complex viscosities of the blends with 20 and 30 wt% PEGMA are highly increased compared with the values expected from the rule of mixtures. In particular, for the blend with 30 wt% PEGMA (TLCP-E30), the shear moduli and complex viscosities are far higher than those of neat PEGMA. In addition, for the blends with high PEGMA contents of 20–30 wt%, tan ™ peaks are observed at a lower frequency range, as can be seen in Figure 9D. These results are believed to be due to the fact that PEGMA-*g*-TLCP copolymers formed during the masterbatch-based melt-compounding contribute to enhancing the compatibility of TLCP and PEGMA components in the blend and restricting the alignment of TLCP chains with the applied shear. 

### 3.4. Dynamic Mechanical Property

**Figure 10:**
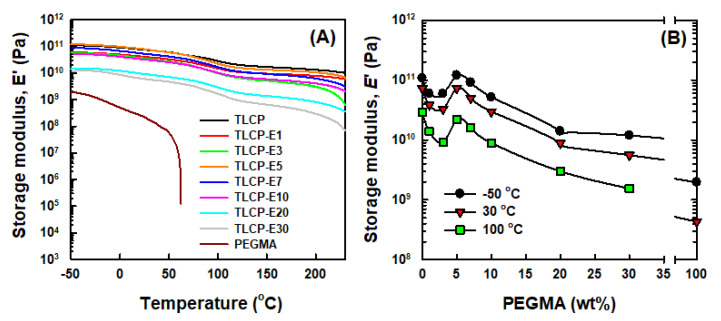
(**A**) Storage modulus vs. temperature and (**B**) storage modulus vs. PEGMA content curves of TLCP, PEGMA, and TLCP/PEGMA blends.

To characterize the effects of the PEGMA content on the dynamic mechanical thermal properties of TLCP/PEGMA blends, the changes of the storage modulus (*E*’) of neat TLCP, neat PEGMA, and their blends as a function of the temperature were obtained, as shown in Figure 10A. For neat TLCP, the storage modulus of ~10^11^ Pa at −50 °C decreased slightly to ~10^10^ Pa at ~100 °C owing to the glass transition, and remained at a level of ~10^10^ Pa up to 230 °C. In the case of neat PEGMA, the storage modulus of ~10^9^ Pa at −50 °C decreased when increasing the temperature and decreased significantly above 50 °C owing to the beginning of the melting of PE crystals. The overall temperature-dependent storage modulus changes of the blends with 1–30 wt% PEGMA are almost identical to the neat TLCP, although the storage moduli are slightly lower over the temperature range.

For a quantitative comparison, the storage moduli of neat TLCP, neat PEGMA, and TLCP/PEGMA blends at constant temperatures of −50, 30, and 100 °C were plotted as a function of the PEGMA content, as shown in Figure 10B. It was found that the storage moduli of the blends at three different temperatures decreased slightly with the PEGMA contents up to 3 wt%, increased at the PEGMA content of 5 wt%, and decreased again at PEGMA contents above 7 wt%. It is believed that the increased storage moduli for the blend with 5 wt% PEGMA loading stem from the dominant reinforcing effects of PEGMA-*g*-TLCP copolymers on the compatibility and mechanical performance of TLCP/PEGMA blends.

## 4. Conclusions

In summary, TLCP-based blends with 1–30 wt% PEGMA loadings were fabricated by facile and efficient masterbatch-based melt-compounding, and their morphology, microstructures, and physical properties were investigated systematically by considering the chemical reaction and intermolecular interaction between TLCP and PEGMA components. The SEM images of cryogenic fracture surfaces revealed that microfibrils of the TLCP component with stiff backbone chains were dispersed uniformly for all blends, without exhibiting any phase-separated domains, which indicates a good compatibility between TLCP and PEGMA components. From the FT-IR spectra, the characteristic vibrational bands associated with the carboxyl groups of TLCP components were found to shift to higher wavenumbers when increasing the PEGMA content in the blends. X-ray diffraction patterns showed that the crystalline diffraction intensity of the PEGMA component in the blends decreased more significantly compared with that of the TLCP component. The morphological and microstructural features obtained from SEM, FT-IR, and XRD data suggested the presence of specific intermolecular interactions between carboxyl/hydroxy groups of TLCP and glycidyl methacrylate groups of PEGMA, as well as the formation of PEGMA-*g*-TLCP copolymers during the melt-compounding. As the results show, the melting and crystallization temperatures of the PEGMA component in the blends were greatly lowered compared to the TLCP component with a rigid backbone chain. The thermal decomposition peak temperatures of PEGMA and TLCP components in the blends were higher than those of neat PEGMA and neat TLCP, respectively. The shear moduli and complex viscosities for the blend with 30 wt% PEGMA at a melt state of 300 °C were far higher than those of neat PEGMA, which supports the presence of PEGMA-*g*-TLCP formed during the melt-compounding. The DMA results revealed that the storage moduli of TLCP/PEGMA blends at a constant temperature decreased slightly with the PEGMA content up to 3 wt%, increased at the PEGMA content of 5 wt%, and decreased again at PEGMA contents above 7 wt%. The maximum storage moduli for the blend with 5 wt% PEGMA were also conjected to be due to the reinforcing and synergistic effects of PEGMA-*g*-TLCP copolymers on the compatibility and mechanical performance of TLCP/PEGMA blends. Overall, it is valid to contend that TLCP/PEGMA blends with enhanced thermal and mechanical properties, as well as an increased price-performance ratio, could be utilized as advanced engineering materials for high-end applications in automotive components, electronic devices, and super fibers.

## Figures and Tables

**Table 1:** Characteristic vibrational bands of neat TLCP, neat PEGMA, and TLCP/PEGMA blends in Fourier-transform infrared (FT-IR) spectra.

Sample Code	Vibrational Band (cm^−1^)		
	$\nu_{c–o,\;\mathrm{sym}}$	$\nu_{c–o,\;\mathrm{asym}}$	$\nu_{c=o,\;\mathrm{carboxyl}}$
TLCP	1176	1249	1724
TLCP-E1	1176	1249	1724
TLCP-E3	1179	1249	1725
TLCP-E5	1179	1249	1724
TLCP-E7	1179	1249	1724
TLCP-E10	1179	1249	1724
TLCP-E20	1179	1251	1725
TLCP-E30	1179	1253	1727
PEGMA	-	1234	1733

**Table 2:** Characteristic melting and crystallization transition temperatures and enthalpies of neat TLCP, neat PEGMA, and TLCP/PEGMAr blends in differential scanning calorimetry (DSC) thermograms.

Sample Code	1st Heating		1st Cooling		2nd Heating	
	*T*_m, PEGMA_/Δ*H*_m, PEGMA_ (°C, J/g)	*T*_m, TLCP_/Δ*H*_m, TLCP_ (°C, J/g)	*T*_c, PEGMA_/Δ*H*_c, PEGMA_ (°C, J/g)	*T*_c, TLCP_/Δ*H*_c, TLCP_ (°C, J/g)	*T*_m, PEGMA_/Δ*H*_m, PEGMA_ (°C, J/g)	*T*_m, TLCP_/Δ*H*_m, TLCP_ (°C, J/g)
TLCP	-	281/43.9	-	237/17.9	-	282/16.1
TLCP-E1	85/1.6	276/1.4	77/0.7	235/4.0	93/0.6	276/1.7
TLCP-E3	93/1.7	276/1.2	79/0.8	237/3.0	92/0.7	277/1.6
TLCP-E5	92/2.5	279/1.1	76/1.9	236/2.1	92/2.1	278/1.5
TLCP-E7	93/4.7	279/1.1	76/3.1	236/2.1	91/3.2	277/1.5
TLCP-E10	95/5.6	278/0.6	76/4.7	237/2.0	92/4.5	277/1.0
TLCP-E20	95/18.2	277/0.5	77/15.5	237/1.7	93/18.3	277/0.7
TLCP-E30	101/29.4	277/0.3	77/27.0	237/1.1	95/26.6	277/0.4
PEGMA	110/448.6	-	90/472.5	-	107/486.4	-

## References

[1] Economy J., Goranov K. (1994). Thermotropic Liquid-Crystalline Polymers for High-Performance Applications. High Performance Polymers.

[2] Jin J.-I. (1994). Microchemical structures and properties of main chain thermotropic polyesters. Mol. Cryst. Liq. Cryst. Sci. Technol. Sec. A Mol. Cryst. Liq. Cryst..

[3] Kwon Y.-W., Choi D.H., Jin J.-I. (2005). Liquid crystalline aromatic polyesters. Polym. Korea.

[4] Yoo T.J., Song B.S., Lee Y.E., Bae B.-S., Jeong Y.G. (2018). Effects of plasticizer on structures, non-isothermal crystallization, and rheological properties of polyarylates. J. Appl. Polym. Sci..

[5] De Kort G.W., Leone N., Stellamanns E., Auhl D., Wilsens C.H.R.M., Rastogi S. (2018). Effect of shear rate on the orientation and relaxation of a vanillic acid based liquid crystalline polymer. Polymers.

[6] Park G.T., Chang J.-H., Lim A.R. (2019). Thermotropic liquid crystalline polymers with various alkoxy side groups: Thermal properties and molecular dynamics. Polymers.

[7] Yamazaki S., Tokita M. (2019). A correlation between thermal diffusivity and long period in thermotropic liquid crystalline polyesters. Macromolecules.

[8] Park G.T., Lee W.J., Chang J.-H., Lim A.R. (2020). Dependence of the physical properties and molecular dynamics of thermotropic liquid crystalline copolyesters on p-hydroxybenzoic acid content. Polymers.

[9] Dutta D., Fruitwala H., Kohli A., Weiss R.A. (1990). Polymer blends containing liquid crystals: A review. Polym. Eng. Sci..

[10] Qin Y., Brydon D.L., Wardman R.H. (1993). Fibres from polypropylene and liquid crystal polymer blends: 3. A comparison of polyblend fibres containing Vectra A900, Vectra B950 and Rodrun LC3000. Polymer.

[11] Datta A., Chen H.H., Baird D.G. (1993). The effect of compatibilization on blends of polypropylene with a liquid-crystalline polymer. Polymer.

[12] Roetting O., Hinrichsen G. (1994). Blends of thermotropic liquid crystalline and thermoplastic polymers: A short review. Adv. Polym. Technol..

[13] Wiff D.R., Weinert R.J. (1998). Blending thermotropic liquid crystal and thermoplastic polymers for microreinforcement. Polymer.

[14] Chen L., Huang H.-Z., Wang Y.-Z., Jow J., Su K. (2009). Transesterification-controlled compatibility and microfibrillation in PC–ABS composites reinforced by phosphorus-containing thermotropic liquid crystalline polyester. Polymer.

[15] Chen G., Chen P., Zeng L., Xu J., Liu P. (2019). Rheological behaviors and properties of poly (ether ether ketone) in-situ blends: Effect of chemical structure of added thermotropic liquid crystalline polyarylates. J. Macromol. Sci. B.

[16] De Kort G.W., Rastogi S., Wilsens C.H.R.M. (2019). Controlling processing, morphology, and mechanical performance in blends of polylactide and thermotropic polyesters. Macromolecules.

[17] Chan C.K., Whitehouse C., Gao P., Chai C.K. (2001). Flow induced chain alignment and disentanglement as the viscosity reduction mechanism within TLCP/HDPE blends. Polymer.

[18] Kim S.H., Park S.W., Gil E.S. (1998). Crystallization kinetics of poly (ethylene terephthalate) with thermotropic liquid crystalline polymer blends. J. Appl. Polym. Sci..

[19] Kim J.Y., Kim S.H. (2005). Structure and property relationship of thermotropic liquid crystal polymer and polyester composite fibers. J. Appl. Polym. Sci..

[20] Chan C.K., Whitehouse C., Gao P. (1999). The effect of TLCP melt structure on the bulk viscosity of high molecular mass polyethylene. Polym. Eng. Sci..

[21] Imai Y., Nishimura S., Abe E., Tateyama H., Abiko A., Yamaguchi A., Aoyama T., Taguchi H. (2002). High-modulus poly(ethylene terephthalate)/expandable fluorine mica nanocomposites with a novel reactive compatibilizer. Chem. Mater..

[22] Kim J.K., Lee H. (1996). The effect of PS-GMA as an in situ compatibilizer on the morphology and rheological properties of the immiscible PBT/PS blend. Polymer.

[23] Kim S., Kim J.K., Park C.E. (1997). Effect of molecular architecture of in situ reactive compatibilizer on the morphology and interfacial activity of an immiscible polyolefin/polystyrene blend. Polymer.

[24] Kudva R.A., Keskkula H., Paul D.R. (1998). Compatibilization of nylon 6/ABS blends using glycidyl methacrylate/methyl methacrylate copolymers. Polymer.

[25] Hale W., Keskkula H., Paul D.R. (1999). Compatibilization of PBT/ABS blends by methyl methacrylate-glycidyl methacrylate-ethyl acrylate terpolymers. Polymer.

[26] Rybnikar F., Yuan B.-L., Geil P.H. (1994). Morphology of nascent melt polymerized poly (2,6-oxynaphthoate/*m*-oxybenzoate) copolymer. Polymer.

[27] Sahre K., Eichhorn K.-J., Reichelt N., Hummel D.O. (1994). Orientational measurements on a crosslinkable thermotropic main-chain copolyester using FTIR spectrometry. Acta Polym..

[28] Chen B.-K., Tsay S.-Y., Chen J.-Y. (2005). Synthesis and properties of liquid crystalline polymers with low Tm and broad mesophase temperature ranges. Polymer.

[29] Shi L.-S., Wang L.-Y., Wang Y.-N. (2006). The investigation of argon plasma surface modification to polyethylene: Quantitative ATR-FTIR spectroscopic analysis. Eur. Polym. J..

[30] Cho K.Y., Eom J.-Y., Kim C.-H., Park J.-K. (2008). Grafting of glycidyl methacrylate onto high-density polyethylene with reaction time in the batch mixer. J. Appl. Polym. Sci..

[31] Undin J., Finne-Wistrand A., Albertsson A.-C. (2013). Copolymerization of 2-methylene-1, 3-dioxepane and glycidyl methacrylate, a well-defined and efficient process for achieving functionalized polyesters for covalent binding of bioactive molecules. Biomacromolecules.

[32] Field N.D., Baldwin R., Layton R., Frayer P., Scardiglia F. (1988). Polymorphism in a liquid crystalline polyester based on 4,4′-bisphenol, terephthalic acid, and p-hydroxybenzoic acid. Macromolecules.

[33] Caminiti R., Pandolfi L., Ballirano P. (2007). Structure of polyethylene from X-ray powder diffraction: Influence of the amorphous fraction on data analysis. J. Macromol. Sci. B.

